# Prognostic Value of Pre-Treatment Prognostic Nutritional Index in Esophageal Cancer: A Systematic Review and Meta-Analysis

**DOI:** 10.3389/fonc.2020.00797

**Published:** 2020-06-17

**Authors:** Jianqi Hao, Cong Chen, Fangfang Wan, Yuzhou Zhu, Hongyu Jin, Jian Zhou, Nan Chen, Jing Yang, Qiang Pu

**Affiliations:** ^1^Department of Thoracic Surgery, West China Hospital, Sichuan University, Chengdu, China; ^2^West China School of Medicine, Sichuan University, Chengdu, China; ^3^Western China Collaborative Innovation Center for Early Diagnosis and Multidisciplinary Therapy of Lung Cancer, Sichuan University, Chengdu, China

**Keywords:** prognostic nutritional index, esophageal cancer, prognosis, meta-analysis, overall survival

## Abstract

**Background:** Prognostic nutritional index (PNI), combining albumin and lymphocyte counts, which represent the nutritional and immune status, was considered as an effective predictor for the patient's prognosis after surgery. To comprehensively analyze the relative effectiveness of prognostic performance of pretreatment PNI in esophageal cancer (EC), we performed this meta-analysis.

**Methods:** We performed a systematic search in PubMed, Embase, CNKI, and Web of Science. The hazard ratios (HRs) or odds ratios (ORs) with their corresponding 95% confidence intervals (CIs) were extracted to explore the correlation between PNI and the post-operative survival of patients with EC, including overall survival (OS), recurrence-free survival (RFS), and post-operative complications. The Newcastle–Ottawa Scale (NOS) was applied to estimate the quality of the included studies. The Begg's test was applied to assess the publication bias.

**Results:** A total of 13 articles with 3,543 patients, were included in our meta-analysis, and nine studies reported OS in 2,731 EC patients. The pooled results of the nine studies suggested that EC patients with a low PNI would have a worse overall survival (HR = 1.14, 95% CI 0.99–1.31, *p* < 0.05). The integrated results also indicated that the PNI was a negative predictor for RFS.

**Conclusion:** This meta-analysis indicated a high correlation between PNI and post-operative survival of EC. EC patients with low PNI values tend to have worse OS and may be at a higher risk of EC recurrence. However, more relevant researches are needed to confirm the association between PNI and post-operative complications of EC.

## Introduction

Esophageal cancer (EC) is a malignant lesion formed by abnormal proliferation of esophageal squamous epithelium or glandular epithelium (1, 2). EC is also the main causative factor of cancer-associated death worldwide, especially in several Eastern and Southern African countries (3). In 2018, it ranked seventh in terms of incidence and sixth in mortality (4). Nowadays, the surgical treatment is still an effective treatment for EC, but the operation for the body is more traumatic, so higher immune level and nutritional status of the patients are required to tolerate the surgical trauma (5). In spite of improvement in the administration and therapies of EC patients, the overall prognosis is still very poor (2). The poor prognosis and rising incidence of EC highlight the need for improved detection and prediction methods. Meanwhile, the detection of genetic biomarkers is very expensive and inconvenient, especially for patients in developing countries (6). Therefore, there is an urgent need to develop easily accessible, cheap, and effective indicators to predict the survival outcome of EC patients, which may help to improve the individualized treatment of EC patients.

Recently, it is widely accepted that cancer patient survival is determined not only by tumor pathology but also by host factors, such as the preoperative nutritional and immunological status (7). Numerous researchers showed that nutrition and immune status were closely associated with tumor progression and prognosis, including the EC (8–14). Among them, the prognostic nutritional index (PNI), calculated by combining the serum albumin levels and the total circulating lymphocyte counts (15), can be an effective indicator for reflecting nutritional and immunological status of cancer patients. Many potential mechanisms could explain this. Albumin is an essential element for cancer patients in their nutrition transport and support especially in their body metabolism (16). Lymphocytes, originating from hematopoietic cells, which include B and T lymphocytes and their effector cells, are the key components of the adaptive immune response (17). Distinct from a single predictor, PNI combines albumin and lymphocyte counts, which represent the nutritional and immune status, can better predict the patient's prognosis after surgery (18). More importantly, the prognostic and clinical value of PNI in different kinds of malignant cancer has been proved with emerging evidence (19–21). Increasingly, studies about the correlation between PNI and prognosis of EC are being conducted. However, most of these studies were performed with a small sample size, and their results remain conflicting. Thus, we conducted this meta-analysis to provide more comprehensive evidence that could confirm the significant prognostic performance of PNI in EC.

## Materials and Methods

This systemic review and meta-analysis were conducted in accordance to the PRISMA statement (22).

### Literature Research

Two independent reviewers (FF and JQ) performed a systemic search in PubMed, Embase, Web of Science, and CNKI using strategies as follows: “prognostic nutritional index(abstract/title),” and “cancer or tumor or carcinoma or neoplasm or malignant or malignancy or adenocarcinoma(abstract/title),” and “esophageal or esophagus(abstract/title).” All available studies were published up to July 2019 without other special limitations, if any discordance happened, we resolved it by consensus.

In addition to searching online and in order to identify potentially eligible articles, we also hand-searched the bibliographies of review articles.

### Selection Criteria and Exclusion Criteria

As a systematic analysis, articles like single sample experiment, comments, case report, letters, review articles, and editorials were eliminated from the study. Finally, the remaining studies were carefully selected when meeting the significant criteria as follows: (a) using pathological diagnoses as a golden standard to confirm the EC; (b) the articles report the hazard ratios (HRs) or odds ratios (ORs) for evaluating the correlations of PNI and overall survival (OS), progression-free survival (PFS), or complications; (c) with full-text accessible. Any disagreement in study selection was resolved by discussion with a third reviewer.

Exclusion criteria: (a) studies that involved non-human subjects, (b) studies with no relevant data on prognostic performance, (c) studies did not provide PNI and HR or OR to evaluate the correlation between PNI and patient survival. If a potential discrepancy was detected, a blinded third reviewer was assigned to adjudicate the conflict.

### Data Extraction and Quality Assessments

The parameters were extracted by two independent investigators (JQ and CC) with a standard extraction table. The listed information of the essays extracted included basic information like title, author, nationality, department, ethnicity, study design, age and gender of the samples, enrolled year, cut-off value, follow-up, management, survival analysis method, HRs, and their 95% confidence intervals (CIs) for OS, PFS, and complications.

The Newcastle–Ottawa Scale (NOS) was used to assess the quality of the included studies (23). We adopted the NOS for the quality of the cohort. The NOS rating can range from 0 to 9, and a study which received a score >7 was considered to be of high quality.

### Statistical Analysis

Statistical analysis was performed by STATA 12.0. The relationship between the prognosis of EC patients and PNI was assessed by HR (95% CI). Heterogeneity across studies was evaluated by χ^2^ and I^2^ test. An I^2^ value of more than 50% indicated significant heterogeneity existed, where random effect model was adopted to pool data. Otherwise, fixed effect model was adopted. A value of HR (low/high PNI) more than 1 with upper limit of its 95% overlap 1 suggested low PNI was a risk of worse survival.

The stoutness of the pooled HR for OS in EC patients was validated by sensitivity analysis. Publication bias was assessed by Begg's test. A *p*-value of < 0.05 suggested significant publication bias, where the influence of publication bias on the merged HR was assessed by trim-and-fill method (24).

## Results

### Search Results

**Figure 1:**
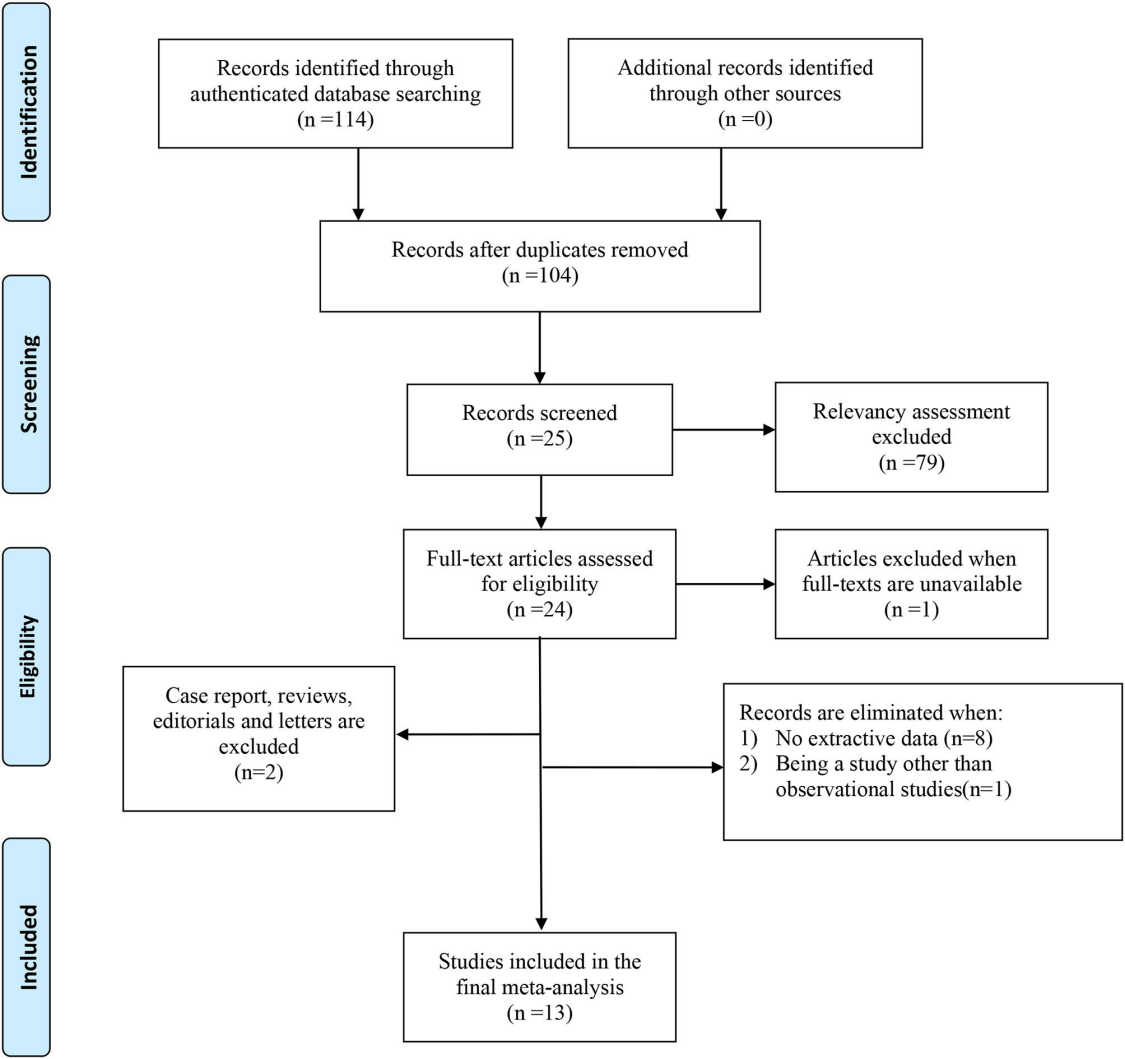
PRISMA 2009 flow diagram. From Moher et al. (25). For more information, visit www.prisma-statement.org.

The process of literature selection and screening was presented in Figure 1. We identified a total of 114 articles through authenticated database searching. And we reviewed 104 abstracts of the studies after excluding 10 duplicate articles. Twenty-five articles were further reviewed in detail after excluding 79 articles on the basis of the relevancy assessment. Of these, one paper was excluded because of unavailability of full text; 24 full-text articles were included for eligibility. Subsequently, we eliminated papers with no extractive data (8), not observational study (1), and other types of articles including review and case report (2). Thirteen articles were included finally.

### Basic Information of the Included Studies

The basic information of the 13 eligible studies (26–38) was presented in Table 1. These 13 studies were published between 2002 and 2018, and the accrual period of these studies was within the 1991–2017 range which had covered a long time span. Among the involved studies, four were performed in China, seven were conducted in Japan, one study was performed in Netherlands, and one was performed in Italy. A total of 3,543 patients with a median age ranging from 61.8 to 67.1 were involved in these studies. The histological type of these studies included squamous carcinoma and adenocarcinoma with a covering TNM stage from I to IV. Four of these studies did not provide the HRs for OS (26–28, 37) but provide the ORs for post-operative complications instead. Two of these articles provided HRs for OS and recurrence-free survival (RFS) (33, 34). Additionally, based on the NOS quality assessment, all the involved studies had a score of 7 or more and one had a score of 8 typically, indicating high quality. The NOS quality assessment of these studies was shown in Table 2.

**Table 1:** The main characteristics of the included studies.

**Study**	**Country**	**Accrual periods**	**Age (median)**	**Number of patients**	**Gender Male/female**	**Histological type**	**TNM stage**	**PNI cut-off value**	**Follow-up (month)**	**Survival data**
Li et al. (20)	China	March 2016– December 2017	65.8	154	107/47		I–III	45		Post-operative complications
Nozoe et al. (27)	Japan	January 1991– December 1998	62.5	258	221/37		I–IV	46	32	Post-operative complications
Han-Geurts et al. (28)	Netherlands	1996–2003	62	400	314/86			42.8		Post-operative complications
Miyazaki et al. (29)	Japan	February 2004– November 2014	65.8	192	173/19		I–IV	47.7	26.5	OS
Ohira et al. (30)	Japan	April 2000– April 2013	63	91	74/17	Squamous carcinoma	IV	40		OS
Fang et al. (38)	China	November 2016– November 2017	61.8	103	81/22	Squamous carcinoma	I–IV	44.56		OS
Chen et al. (31)	China	January 2005– December 2008		308	268/40		I–III	45		OS
Zhang et al. (32)	China	October 2010– December 2011	64	355	281/74	Adenocarcinoma (Siewert type II/III)	I–IV	51.3	52	OS
Urabe et al. (33)	Japan	February 1999– September 2014		1,363	974/389	Adenocarcinoma	I–IV	44.8	63.3	OS and RFS
Nakatani et al. (34)	Japan	January 2009– August 2015	64.7	66	56/10	Squamous carcinoma	I–IV	45		OS and RFS
Matsumoto et al. (35)	Japan	April 2010– May 2015	66.5	84	77/7		I–IV	45	34.6	OS
Hirahara et al. (36)	Japan	January 2006– December 2015	67.1	169	150/19	Squamous carcinoma	II–IV	49.2		OS
Filip et al. (37)	Italy	January 2008– October 2012	62	167	137/30	Adenocarcinoma (Siewert type I and II)	I–IV			Post-operative complications

*OS, overall survival; RFS, recurrence-free survival*.

**Table 2:** The Newcastle-Ottawa Scale (NOS) quality assessment of the eligible studies.

**Study**			**Selection**				**Comparability**	**Outcome**			**Total**
			**Representativeness of the exposed cohort**	**Selection of the non-exposed cohort**	**Ascertainment of exposure**	**Demonstration that outcome of interest was not present at start of study**	**Comparability of cohorts on the basis of the design or analysis**	**Assessment of outcome**	**Was follow-up long enough for outcomes to occur**	**Adequacy of follow up of cohorts**	
Chen et al. (31)	308	268/40	*	*		*	*	*	*	*	7
Fang et al. (38)	103	81/22	*	*	*	*	*		*	*	7
Filip et al. (37)	167	137/30	*	*	*		*	*	*	*	7
Han-Geurts et al. (28)	400	314/86	*	*	*	*		*	*	*	7
Hirahara et al. (36)	169	150/19	*	*	*	*	*	*		*	7
Li et al. (20)	154	107/47	*	*	*	*	*	*	*	*	8
Matsumoto et al. (35)	84	77/7		*	*	*	*	*	*	*	7
Miyazaki et al. (29)	192	173/19	*	*	*	*	*		*	*	7
Nakatani et al. (34)	66	56/10		*	*	*	*	*	*	*	7
Nozoe et al. (27)	258	221/37	*	*	*	*		*	*	*	7
Ohira et al. (30)	91	74/17		*	*	*	*	*	*	*	7
Urabe et al. (33)	1363	974/389	*	*	*	*		*	*	*	7
Zhang et al. (32)	355	281/74	*	*	*	*		*	*	*	7

### Pooling Analysis

#### Correlation Between Prognostic Nutritional Index and Overall Survival in Esophageal Cancer Patients

**Figure 2:**
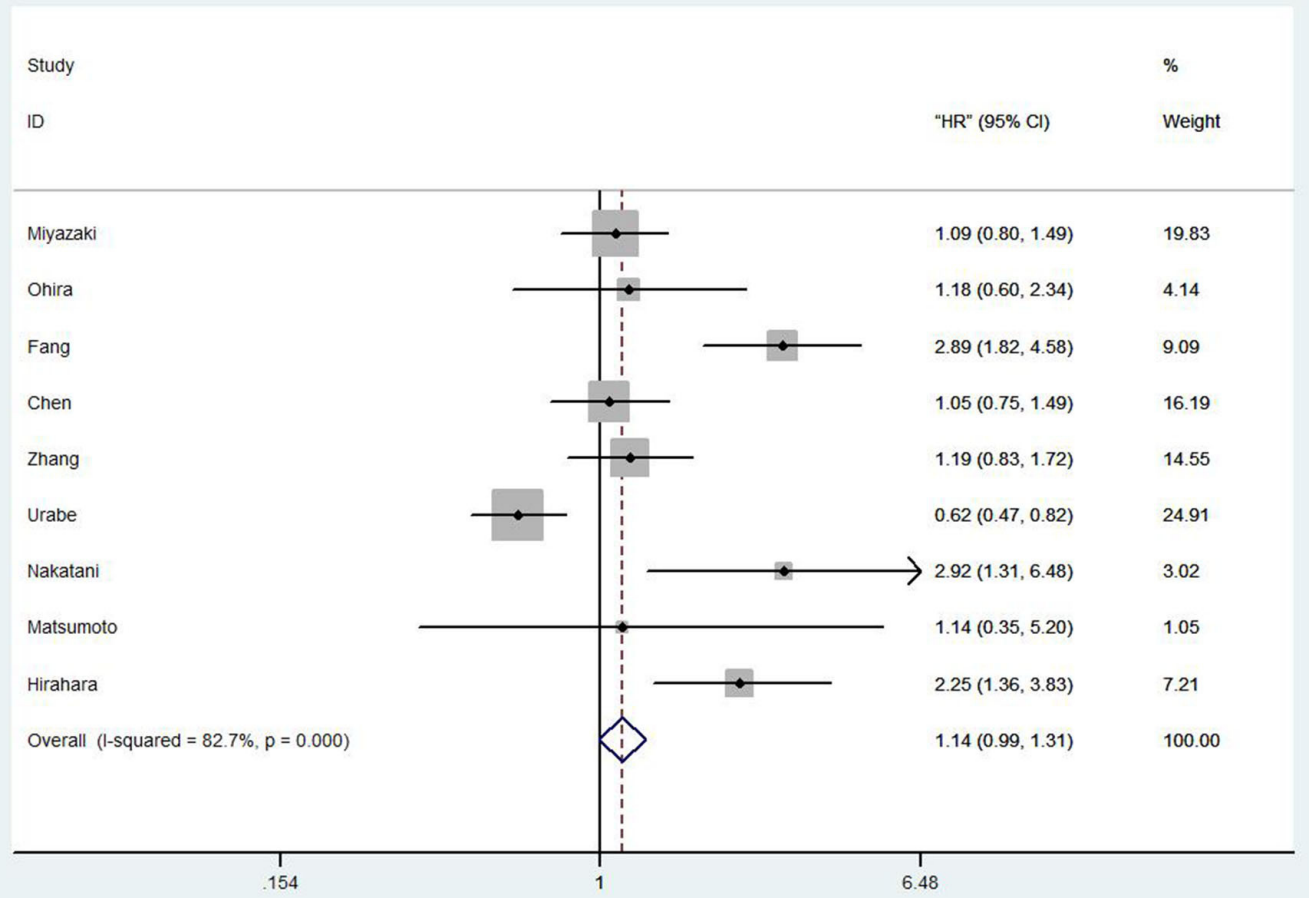
Forest plot of the HR assessing the correlation between the PNI and OS in EC patients. HR, hazard ratio; EC, esophageal cancer; OS, overall survival; PNI, prognostic nutritional index.

Totally, nine studies reported OS in 2,731 EC patients. Our results showed that among the EC patients with low PNI tends to a worse OS (HR 1.14, 95% CI 0.99–1.31, *p* = 0.001; Figure 2).

#### Correlation Between Prognostic Nutritional Index and Recurrence-Free Survival in Esophageal Cancer Patients

Two studies (33, 34) with 1,429 patients provided available data for discussing the correlation between PNI and RFS. Urabe et al. (33) analyzed the data by using univariate Cox regression analysis, and their results showed that preoperative PNI was significantly associated with RFS (HR = 0.60, 95% CI 0.46–0.78, *p* < 0.001). Nakatani et al. (34) also agreed that the preoperative PNI was significantly associated with the RFS (HR = 2.35, 95% CI 1.11–4.96, *p* = 0.026). Although the two studies both suggested that patients with low PNI may tend to have a high post-operation recurrence rate, further researches should be needed in the future.

#### Correlation Between Prognostic Nutritional Index and Post-operative Complications in Esophageal Cancer Patients

**Figure 3:**
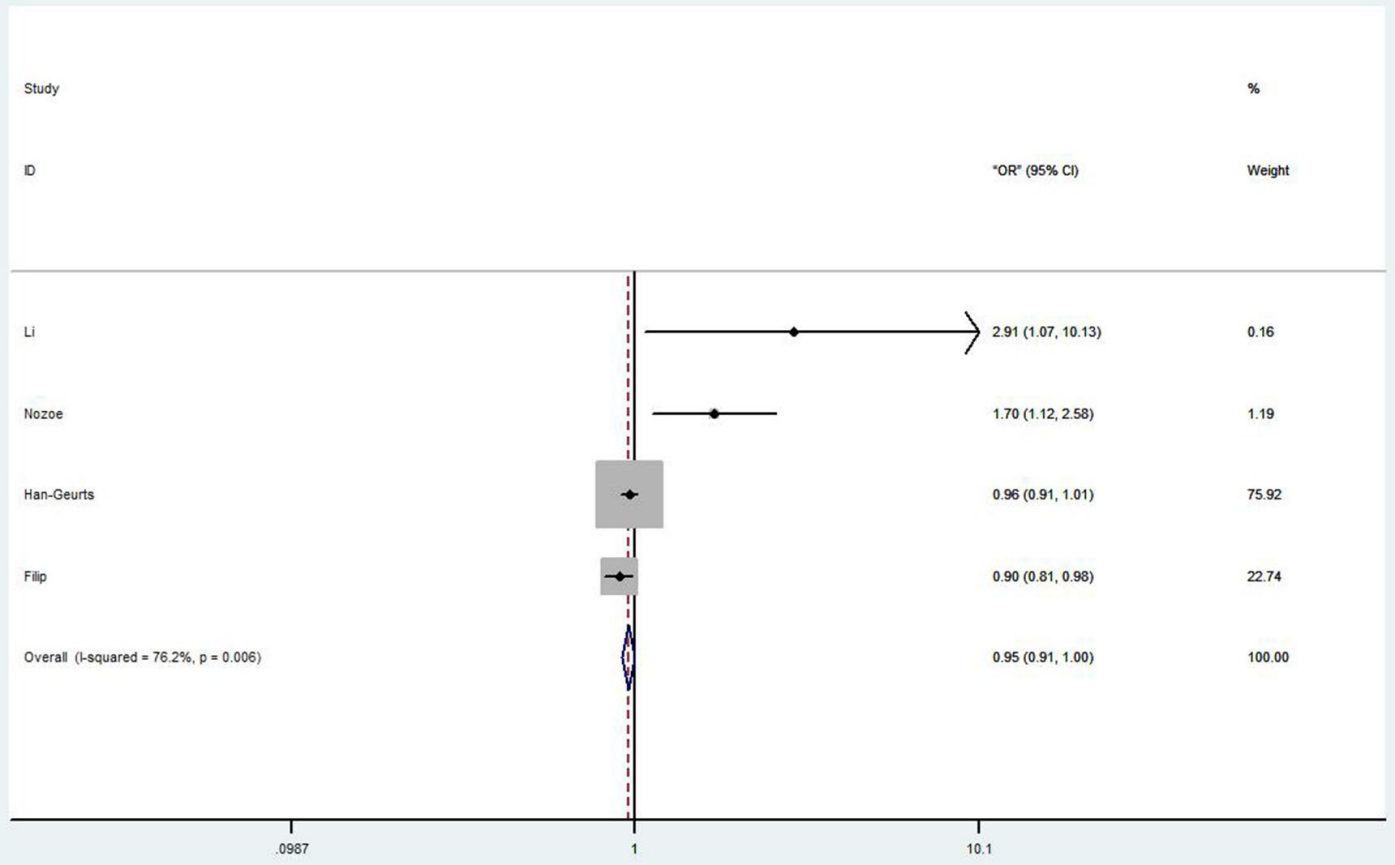
Forest plot of the HR assessing the correlation between the PNI and complications in EC patients. HR, hazard ratio; EC, esophageal cancer; PNI, prognostic nutritional index.

Four studies discussed the correlation between PNI and post-operative complications in EC patients (26–28, 37). Li et al. (26) showed that low PNI was an independent risk factor for overall post-operative complications and severe post-operative complications (OR = 2.31, 95% CI 1.058–6.821, *p* = 0.036; OR = 2.91, 95% CI 1.067–10.131, *p* = 0.040, respectively). Nozoe et al. (27) also agreed that the post-operative complications in patients with higher PNI value were significantly less than that in patients with lower PNI value (OR = 3.50, 95% CI 1.89–6.49, *p* < 0.0001). However, Han-Geurts et al. (28) argued that preoperative PNI had limited value in predicting complications after esophageal resection (OR 0.96, 95% CI 0.91–1.01, *p* = 0.078). Although Filip et al. (37) showed an undesirable result from their study (OR 0.90, 95% CI 0.81–0.98, *p* = 0.04), they still believed PNI could be an independent risk factor for major complications. The overall HR (OR 0.95, 95% CI 0.91–1.00, *p* = 0.001) of these studies was shown in Figure 3.

### Sensitivity Analysis

**Figure 4:**
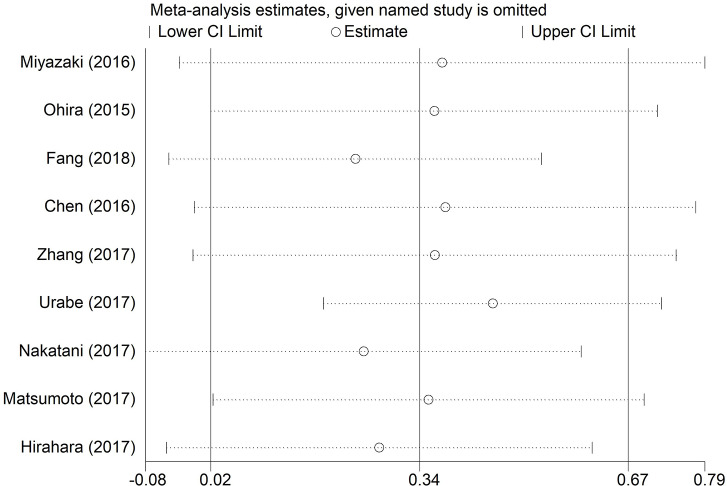
Sensitivity analyses for testing the robustness of the pooled HR for OS in EC patients. HR, hazard ratio; EC, esophageal cancer; OS, overall survival.

The robustness of pooled results of OS in EC patients was assessed through sensitivity analysis in our study. We found that the integrated HRs for OS did not change significantly, revealing the robustness of the pooled results. The result was shown in Figure 4. Because few eligible studies were reported for RFS or post-operative complications in EC patients, we did not perform the sensitivity analysis for RFS and post-operative complications in this analysis.

### Publication Bias

**Figure 5:**
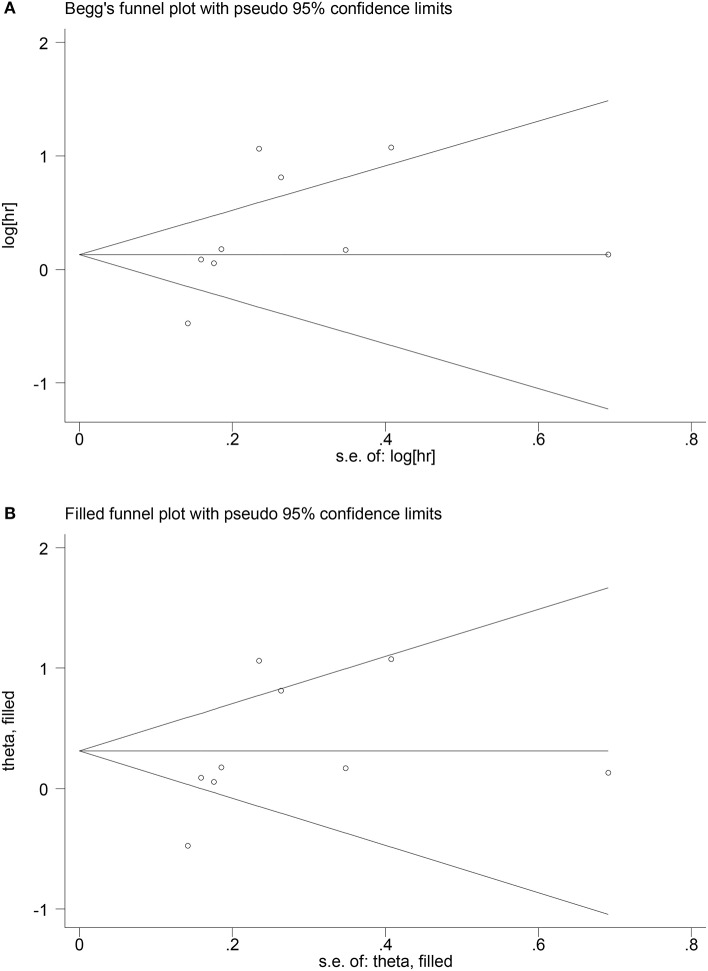
Begg's funnel plot of publication bias for OS **(A)**; adjusted Begg's funnel plot from trim-and-fill analysis for HR for OS in EC patients **(B)**.

The Begg's funnel test and Egger's test were applied to assess the publication bias for OS in EC patients. The funnel plot of the Begg's test showed an asymmetry of the result, and a significant publication bias was also shown in the Egger's test (*p* < 0.05). Subsequently, we used trim-and-fill method to assess the effect of publication bias on the reliability of the integrated HRs for OS (Figure 5). The results showed no trimming was performed for the data, indicating that the publication bias did not essentially affect the reliability of the integrated HRs for OS. As for the correlation between PNI and RFS or post-operative complications, the publication bias was not performed in this meta-analysis because <10 eligible studies were reported.

## Discussion

Totally, 13 studies were considered into our meta-analysis. The pooled results demonstrated that EC patients with low PNI value tend to have worse survival (OS) and a high risk of cancer recurrence rate.

Certain reports have suggested that preoperative PNI is associated with survival after surgery in patients with gastric (39) and colorectal cancer (40).

In general, our results indicated that patients with poor PNI value would have a high risk of post-operation complications which would shorten the OS time of the cancer patients. Moreover, poor nutritional and immune status could make cancer patients unable to tolerate post-operative adjuvant therapy (41). As a consequence, patients abandon post-operative adjuvant therapy, which shorten the survival time and make patients have a poor quality of life. Additionally, Nozoe et al. (27) reported that low PNI also contributed to the tumor progression.

Recently, many researchers have conducted a lot of correlative clinical researches, but so far there are still no comprehensive results. We systematically searched the databases, and then we conducted a comprehensive analysis of the results. Taken together, our pooled results demonstrated that poor PNI value is a high risk for worse prognosis in EC patients after surgery. Our meta-analysis suggested that nutritional status can be assessed by PNI, and if necessary, nutritional support treatment should be actively given before surgery.

According to the forest plot of the HR assessing the correlation between the PNI and complications in EC patients, the overall result was <1, which suggested low PNI was not a risk factor of the complications after surgery. Then we conducted a careful analysis of the results. We found that in Han et al. (42), they reported that PNI had a limited value in predicting complications following esophageal resection with the OR (OR = 0.96, 95% CI 0.91–1.01, *p* = 0.078). In Filip et al. (43), they did a multivariable analysis between PNI and major complications; they found that the OR was 0.90 (95% CI 0.81–0.90, *p* = 0.04), so they come to a conclusion that nutritional status as PNI was significantly related with the occurrence of severe complications.

On the contrary, Li et al. (26) and Nozoe et al. (27) doubtlessly reported that low PNI patients with poor PNI value would have a high risk of post-operation complications. And the ORs of the complications were 2.91 (95% CI 1.067–10.131, *p* = 0.04) and 1.70 (95% CI 1.12–2.58, *p* = 0.012), respectively. There were few researches on PNI in predicting post-operative complications in patients with EC. And Han's research has a larger weight in the forest plot, which affects the comprehensive analysis of the results, and may affect the reliability of the results. More well-designed relevant researches are required in the future to confirm the predictive value of PNI in post-operative complications of EC.

There have been two other meta-analyses published on the related topic before. Li et al. (44) conducted a meta-analysis with nine articles about the prognostic value of pretreatment PNI in esophageal squamous cell carcinoma. And their conclusion was that low pretreatment PNI was significantly related with OS and RFS. PNI was a reliable prognostic factor of EC, and higher stage EC had higher incidence of low PNI. Their report confirmed our research. However, their studies only focus on the esophageal squamous cell carcinoma, our research also proved the prognostic value of PNI in other types of EC (such as esophageal adenocarcinoma). In addition, in the analysis of esophageal squamous cell carcinoma, the articles we included were not exactly the same, so our conclusions are further confirmed.

Liao et al. (45) conducted a meta-analysis with 12 articles. They also reported that the lower PNI was correlated with unfavorable prognostic factor and poor prognosis in patients with EC. However, their included studies were all from eastern Asia countries; therefore, ethnic bias may exist. Our researches included Italy and Netherlands studies; this may reduce ethnic bias and make the result more reliable. Moreover, the articles we included were not exactly the same, so our conclusions are further confirmed again.

This meta-analysis has some clinical implications. Lower PNI is noteworthily associated with worse OS in EC after surgery. However, there are still many limitations ought to be considered before clinical implications. Firstly, among the included studies, the cut-off value of PNI ranged from 40 to 51.3. Most studies set the cut-off value as 45. A PNI value >45 defined as high PNI and <45 defined as low PNI. The cut-off values of PNI were not consistent in all the included studies, hence a consistent optimal cut-off value of PNI is needed to be determined before PNI is extensively applied for predicting the prognosis of EC patients and their clinical treatment decision. Secondly, few researches were conducted on the association between PNI and complications, whether the PNI has a good prediction performance of complications remains unknown and future well-designed researches are required to demonstrate this issue. Thirdly, since almost all of the researches were conducted in Asia, it might generate selection bias. Fourthly, since we could not obtain the more detailed original data in the included studies, we did not perform a specific subgroup analysis on the base of stage, race, surgical approach, and so on.

## Conclusion

This meta-analysis indicated a high correlation between PNI and post-operative survival of EC. EC patients with low PNI value tend to have worse OS and may be at a higher risk of EC recurrence. However, more relevant researches are needed to confirm the association between PNI and post-operative complications of EC.

## Data Availability Statement

The original contributions presented in the study are included in the article/supplementary material, further inquiries can be directed to the corresponding author puqiang100@163.com.

## Author Contributions

QP and JH contributed to the protocol/project development. CC and FW contributed to the data collection or management. CC, NC, and HJ contributed to the data analysis. JZ and JY contributed to the investigation. QP contributed to the funding acquisition. YZ was in charge of the software. JH contributed to writing the original draft. QP and JH contributed to the manuscript writing and review and editing. All authors contributed to the article and approved the submitted version.

## Conflict of Interest

The authors declare that the research was conducted in the absence of any commercial or financial relationships that could be construed as a potential conflict of interest.

## Abbreviations

CIs: confidence intervals
HRs: hazard ratios
OR: odds ratio
ORs: odds ratios
OS: overall survival
PNI: prognostic nutritional index
EC: esophageal cancer
RFS: recurrence-free survival.

## References

[1] Enzinger PC Mayer RJ. Esophageal cancer. N Engl J Med. (2003) 349:2241–52. 10.1056/NEJMra035010 14657432

[2] Huang FL Yu SJ. Esophageal cancer: risk factors, genetic association, and treatment. Asian J Surg. (2018) 41:210–5. 10.1016/j.asjsur.2016.10.005 27986415

[3] Domper Arnal MJ Ferrandez Arenas A Lanas Arbeloa A. Esophageal cancer: risk factors, screening and endoscopic treatment in Western and Eastern countries. World J Gastroenterol. (2015) 21:7933–43. 10.3748/wjg.v21.i26.7933 26185366 PMC4499337

[4] Bray F Ferlay J Soerjomataram I Siegel RL Torre LA Jemal A. Global cancer statistics 2018: GLOBOCAN estimates of incidence and mortality worldwide for 36 cancers in 185 countries. CA Cancer J Clin. (2018) 68:394–424. 10.3322/caac.21492 30207593

[5] Kato H Nakajima M. Treatments for esophageal cancer: a review. Gen Thorac Cardiovasc Surg. (2013) 61:330–5. 10.1007/s11748-013-0246-0 23568356

[6] Wang Z Wang Y Zhang X Zhang T. Pretreatment prognostic nutritional index as a prognostic factor in lung cancer: review and meta-analysis. Clin Chim Acta. (2018) 486:303–10. 10.1016/j.cca.2018.08.030 30138620

[7] Schwegler I von Holzen A Gutzwiller JP Schlumpf R Muhlebach S Stanga Z. Nutritional risk is a clinical predictor of postoperative mortality and morbidity in surgery for colorectal cancer. Br J Surg. (2010) 97:92–7. 10.1002/bjs.6805 20013933

[8] Park S Lee SH Suh B Keam B Kim TM Kim DW. Nutritional status in the era of target therapy: poor nutrition is a prognostic factor in non-small cell lung cancer with activating epidermal growth factor receptor mutations. Korean J Intern Med. (2016) 31:1140–9. 10.3904/kjim.2015.062 27017943 PMC5094922

[9] Fan N Chen D Zheng J Wen Z Lin P. A novel preoperative plasma indicator to predict prognoses for patients with esophageal squamous cell carcinoma after radical esophagectomy: fibrinogen-to-lymphocyte ratio. Cancer Manag Res. (2019) 11:4719–28. 10.2147/CMAR.S204938 31213896 PMC6536709

[10] Okada G Momoki C Habu D Kambara C Fujii T Matsuda Y. Effect of postoperative oral intake on prognosis for esophageal cancer. Nutrients. (2019) 11:1338. 10.3390/nu11061338 31207910 PMC6627190

[11] Qiu Y You J Lv Q Yuan L Effect of whole-course nutrition management on patients with esophageal cancer undergoing concurrent chemoradiotherapy: a randomized control trial (P05-031-19). Curr. Dev. Nutr. (2019) 3:nzz030P05-031-19. 10.1093/cdn/nzz030.P05-031-19 31526964

[12] Song Q Wu JZ Wang S. Low preoperative lymphocyte to monocyte ratio serves as a worse prognostic marker in patients with esophageal squamous cell carcinoma undergoing curative tumor resection. J Cancer. (2019) 10:2057–62. 10.7150/jca.29383 31205566 PMC6548163

[13] Zhang X Hu D Lin X Zhang H Xia Y Lin J. Prognostic value of an inflammation-related index in 6,865 Chinese patients with postoperative digestive tract cancers: the FIESTA study. Front Oncol. (2019) 9:427. 10.3389/fonc.2019.00427 31192131 PMC6538942

[14] Gervais C Boudou-Rouquette P Jouinot A Huillard O Alexandre J Arrondeau J Predictive and prognostic value of systemic inflammatory response biomarkers in patients receiving nivolumab for metastatic non-small cell lung cancer (NSCLC). J Clin Oncol Conf. (2017) 35:3055 10.1200/JCO.2017.35.15_suppl.3055

[15] Okadome K Baba Y Yagi T Kiyozumi Y Ishimoto T Iwatsuki M. Prognostic nutritional index, tumor-infiltrating lymphocytes, and prognosis in patients with esophageal cancer. Ann Surg. (2018) 271:693–700. 10.1097/SLA.0000000000002985 30308614

[16] Infusino I Panteghini M. Serum albumin: accuracy and clinical use. Clin Chim Acta. (2013) 419:15–8. 10.1016/j.cca.2013.01.005 23348571

[17] Zhao W Wang P Jia H Chen M Gu X Liu M Lymphocyte count or percentage: which can better predict the prognosis of advanced cancer patients following palliative care? BMC Cancer. (2017) 17:514 10.1186/s12885-017-3498-8 28768490 PMC5541405

[18] Hu Y Shen J Liu R Feng Z Zhang C Ling L. Prognostic value of pretreatment prognostic nutritional index in non-small cell lung cancer: a systematic review and meta-analysis. Int J Biol Markers. (2018) 33:372–8. 10.1177/1724600818799876 30282502

[19] Sun K Chen S Xu J Li G He Y. The prognostic significance of the prognostic nutritional index in cancer: a systematic review and meta-analysis. J Cancer Res Clin Oncol. (2014) 140:1537–49. 10.1007/s00432-014-1714-3 24878931 PMC11823704

[20] Li D Yuan X Liu J Li C Li W. Prognostic value of prognostic nutritional index in lung cancer: a meta-analysis. J Thorac Dis. (2018) 10:5298–307. 10.21037/jtd.2018.08.51 30416777 PMC6196222

[21] Yang Y Gao P Song Y Sun J Chen X Zhao J. The prognostic nutritional index is a predictive indicator of prognosis and postoperative complications in gastric cancer: a meta-analysis. Eur J Surg Oncol. (2016) 42:1176–82. 10.1016/j.ejso.2016.05.029 27293109

[22] Shamseer L Moher D Clarke M Ghersi D Liberati A Petticrew M Preferred reporting items for systematic review and meta-analysis protocols (PRISMA-P) 2015: elaboration and explanation. BMJ. (2016) 354:i4086 10.1136/bmj.g7647 27444514

[23] Stang A. Critical evaluation of the Newcastle-Ottawa scale for the assessment of the quality of nonrandomized studies in meta-analyses. European journal of epidemiology. (2010) 25:603-605. 10.1007/s10654-010-9491-z 20652370

[24] Duval S Tweedie R. Trim and fill: a simple funnel-plot-based method of testing and adjusting for publication bias in meta-analysis. Biometrics. (2000) 56:455–63. 10.1111/j.0006-341X.2000.00455.x 10877304

[25] Moher D Liberati A Tetzalff J Altman DG The PRISMA Group Preferred reporting items for systematic reviews and meta-analyses: the PRISMA statement. PLos Med. (2009) 6:e1000097 10.1371/journal.pmed.1000097 19621072 PMC2707599

[26] Li M Yang S Huang X Predictive value of prognostic nutritional index for postoperative complications of esophageal cancer. J Clin Pathol Res. (2018) 38:1267–73.

[27] Nozoe T Kimura Y Ishida M Saeki H Korenaga D Sugimachi K. Correlation of pre-operative nutritional condition with post-operative complications in surgical treatment for oesophageal carcinoma. Eur J Surg Oncol. (2002) 28:396–400. 10.1053/ejso.2002.1257 12099649

[28] Han-Geurts IJ Hop WC Tran TC Tilanus HW. Nutritional status as a risk factor in esophageal surgery. Dig Surg. (2006) 23:159–63. 10.1159/000093756 16888387

[29] Miyazaki T Sakai M Sohda M Tanaka N Yokobori T Motegi Y. Prognostic significance of inflammatory and nutritional parameters in patients with esophageal cancer. Anticancer Res. (2016) 36:6557–62. 10.21873/anticanres.11259 27919983

[30] Ohira M Kubo N Masuda G Yamashita Y Sakurai K Toyokawa T. Glasgow prognostic score as a prognostic clinical marker in T4 esophageal squamous cell carcinoma. Anticancer Res. (2015) 35:4897–901. 26254385

[31] Chen S Yang X Feng JF. A novel inflammation-based prognostic score for patients with esophageal squamous cell carcinoma: the c-reactive protein/prognostic nutritional index ratio. Oncotarget. (2016) 7:62123–32. 10.18632/oncotarget.11389 27557504 PMC5308715

[32] Zhang L Su Y Chen Z Wei Z Han W Xu A. The prognostic value of preoperative inflammation-based prognostic scores and nutritional status for overall survival in resected patients with nonmetastatic Siewert type II/III adenocarcinoma of esophagogastric junction. Medicine. (2017) 96:0000000000007647. 10.1097/MD.0000000000007647 28746229 PMC5627855

[33] Urabe M Yamashita H Watanabe T Seto Y. Comparison of prognostic abilities among preoperative laboratory data indices in patients with resectable gastric and esophagogastric junction adenocarcinoma. World J Surg. (2018) 42:185–94. 10.1007/s00268-017-4146-9 28741195

[34] Nakatani M Migita K Matsumoto S Wakatsuki K Ito M Nakade H. Prognostic significance of the prognostic nutritional index in esophageal cancer patients undergoing neoadjuvant chemotherapy. Dis Esophagus. (2017) 30:1–7. 10.1093/dote/dox020 28575242

[35] Matsumoto H Okamoto Y Kawai A Ueno D Kubota H Murakami H. Prognosis prediction for postoperative esophageal cancer patients using Onodera's prognostic nutritional index. Nutr Cancer. (2017) 69:849–54. 10.1080/01635581.2017.1339093 28726497

[36] Hirahara N Tajima Y Fujii Y Kaji S Yamamoto T Hyakudomi R. Preoperative prognostic nutritional index predicts long-term surgical outcomes in patients with esophageal squamous cell carcinoma. World J Surg. (2018) 42:2199–208. 10.1007/s00268-017-4437-1 29290069 PMC5990565

[37] Filip B Scarpa M Cavallin F Cagol M Alfieri R Saadeh L. Postoperative outcome after oesophagectomy for cancer: nutritional status is the missing ring in the current prognostic scores. Eur J Surg Oncol. (2015) 41:787–94. 10.1016/j.ejso.2015.02.014 25890494

[38] Fang Y Zhang R Huang Y Yao L Fang K Application of neutrophil/lymphocyte ratio and prognostic nutrition index in evaluating the prognosis of patients with esophageal squamous cell carcinoma. J An Hui Med School. (2018) 53:1294–8.

[39] Nozoe T Ninomiya M Maeda T Matsukuma A Nakashima H Ezaki T. Prognostic nutritional index: a tool to predict the biological aggressiveness of gastric carcinoma. Surg Today. (2010) 40:440–3. 10.1007/s00595-009-4065-y 20425547

[40] Maeda K Shibutani M Otani H Nagahara H Sugano K Ikeya T. Low nutritional prognostic index correlates with poor survival in patients with stage IV colorectal cancer following palliative resection of the primary tumor. World J Surg. (2014) 38:1217–22. 10.1007/s00268-013-2386-x 24305937

[41] Kurumisawa S Kawahito K. Risk analysis using the prognostic nutritional index in hemodialysis-dependent patients undergoing cardiac surgery. J Artif Organs. (2018) 21:443–9. 10.1007/s10047-018-1056-z 29951931

[42] Han L Song Q Jia Y Chen X Wang C Chen P. The clinical significance of systemic inflammation score in esophageal squamous cell carcinoma. Tumour Biol. (2016) 37:3081–90. 10.1007/s13277-015-4152-1 26423404

[43] Scarpa M Filip B Cavallin F Alfieri R Saadeh L Cagol M. Esophagectomy in elderly patients: which is the best prognostic score? Dis Esophagus. (2016) 29:589–97. 10.1111/dote.12358 25873285

[44] Li P Wang X Lai Y Zhou K Tang Y Che G. The prognostic value of pre-treatment prognostic nutritional index in esophageal squamous cell carcinoma: a meta-analysis. Medicine. (2019) 98:e15280. 10.1097/MD.0000000000015280 31145271 PMC6709023

[45] Liao G Zhao Z Yang H Chen M Li X. Can prognostic nutritional index be a prediction factor in esophageal cancer? A meta-analysis. Nutr Cancer. (2020) 72:187–93. 10.1080/01635581.2019.1631859 31272238

